# NordICC Trial Results in Line With Expected Colorectal Cancer Mortality Reduction After Colonoscopy: A Modeling Study

**DOI:** 10.1053/j.gastro.2023.06.035

**Published:** 2023-07-15

**Authors:** DANICA M.N. VAN DEN BERG, PEDRO NASCIMENTO DE LIMA, AMY B. KNUDSEN, CAROLYN M. RUTTER, DAVID WEINBERG, IRIS LANSDORP-VOGELAAR, THE CISNET-COLON GROUP

**Affiliations:** Department of Public Health, Erasmus MC University Medical Center, Rotterdam, The Netherlands; RAND Corporation, Arlington, Virginia; Institute for Technology Assessment, Department of Radiology, Massachusetts General Hospital, Boston, Massachusetts; Biostatistics Program, Public Health Sciences Division, Fred Hutchinson Cancer Center, Hutchinson Institute for Cancer Outcomes Research, Seattle, Washington; Department of Medicine, Fox Chase Cancer Center, Philadelphia, Pennsylvania; Department of Public Health, Erasmus MC University Medical Center, Rotterdam, The Netherlands

Colonoscopy screening is a widely recommended method for detecting colorectal cancer (CRC) in countries across the world.^1^ However, until recently, no randomized controlled trials demonstrated its effectiveness in average-risk individuals. Recently, Bretthauer et al^2^ published preliminary results of a multicenter randomized controlled trial, the Nordic-European Initiative on Colorectal Cancer (NordICC) trial, that investigated the effects of once-only colonoscopy screening on CRC incidence and mortality.^2^ In the intention-to-screen analysis, which compared participants not offered screening to those offered screening regardless of participation, they found that the invited group had an incidence and mortality reduction at 10 years of 18% and 10%, respectively. The investigators noted that although the incidence and mortality reductions were clinically important, they were lower than anticipated based on observational and modeling studies.

The publication of the NordICC trial results induced media attention and controversy regarding the effectiveness of colonoscopies.^3^ Experts advised people to interpret the results cautiously, noting aspects of the NordICC trial that could contribute to the underwhelming findings. A critical issue was the low screening uptake (42%). In the adjusted per-protocol analyses, which compared participants not offered screening to those offered screening who received colonoscopy, incidence and mortality reductions at 10 years increased to 31% and 50%, respectively. Another important consideration was the relatively short 10-year follow-up period. This study aimed to evaluate whether the NordICC trial results are lower than expected based on modeling and to what extent the results could be explained by screening uptake and follow-up period.

We used 3 Cancer Intervention and Surveillance Modeling Network CRC models to simulate NordICC trial outcomes: Colorectal Cancer Simulated Population Model for Incidence and Natural History (CRCSPIN), Microsimulation Screening Analysis Colorectal Cancer (MISCAN-Colon), and Simulation Model of Colorectal Cancer (SimCRC). Using these models, we simulated the NordICC trial population,^2^ with 42% of the invited group simulated to receive a 1-time colonoscopy and a usual-care group remaining unscreened (Supplementary Table 1). Our modeling assumptions included random selection into screening unrelated to CRC risk, full adherence to US guidelines for adenoma surveillance,^4^ and high sensitivity of colonoscopy (Supplementary Materials). We compared model predictions to reductions in CRC incidence and mortality observed in the trial. Additionally, we simulated 5 hypothetical scenarios: 42% adherence with 15- and 20-year follow-up and 100% adherence with 10-, 15-, and 20-year follow-up.

With 42% uptake and 10-year follow-up, the models predicted CRC incidence and mortality reductions of 11%–28% and 24%–32% (ranges are across models), respectively (Figure 1A and B). These estimates overlap the 95% confidence intervals (Cis) of the decreases observed in the NordICC intention-to-screen analyses, which were 18% (95% CI: 7–30) and 10% (95% CI: −16 to 36), respectively. The level of screening uptake had the largest impact on the findings: with 100% uptake, the model-predicted incidence and mortality reductions more than doubled to 26%–61% and 53%–70%, respectively (Figure 1C and D). These estimates compared well with reductions of 31% (95% CI: 17–45) and 50% (95% CI: 23–73), respectively, in the per-protocol NordICC analyses. Although the relative differences in risk reduction are substantial, the absolute incidence and mortality reduction only increased from 0.14%–0.29% to 0.31%–0.64% and from 0.10%–0.12% to 0.22%–0.26%, respectively, with 42% vs 100% uptake (Supplementary Figure 1).

With 42% uptake, the predicted incidence reduction increased to 18%–33% and 19%–35% at the 15- and 20-year follow-up, respectively (Figure 1A). With 100% uptake, these reductions increased to 40%–73% and 43%–77%, respectively (Figure 1C). Combining 100% uptake with a 15-year follow-up resulted in expected incidence and mortality reductions of 40%–73% and 59%–79%, respectively (Figure 1C and D).

In this study, we show that, in spite of suggestions otherwise,^2^ model predictions are consistent with the NordICC trial results. As experts have pointed out, the results of the NordICC trial are largely determined by the screening uptake and the follow-up duration. Prior observational studies reported that colonoscopy was associated with a pooled CRC mortality reduction of 62% (range, 11%–88%) at an average follow-up of 8 years.^5^ This is within the CI of the per-protocol NordICC trial results and in line with the modeling results, which estimated an average 63% CRC mortality reduction with 100% uptake and the 10-year follow-up.

A limitation of our study is that we assumed similar CRC risk for screening participants and nonparticipants. The trial results could be influenced by the healthy screenee effect, with people participating in screening at lower risk of CRC. If participants have a lower CRC risk than nonparticipants, this means that our models overestimate the effectiveness of screening. On the other hand, NordICC trial results show that the noninvited group had a higher risk of CRC than non-participants in the invited group,^2^ suggesting that there was self-selection of higher-risk individuals participating in screening (eg, those with a family history of CRC or symptoms). A higher CRC risk in participants than nonparticipants implies we may have underestimated screening effectiveness in our models. Systematic differences between screened and unscreened participants in the intervention group might explain differences between trial estimates of CRC mortality and model predictions. A second limitation concerns our assumed colonoscopy sensitivity. If the colonoscopy sensitivity achieved in the trial was lower than the sensitivity assumed when making model projections, then the projected benefits would be optimistic. Lower colonoscopy sensitivity would allow more adenomas to progress to cancer, reducing the effectiveness of colonoscopy.

The trial’s 42% uptake aligns with the 5%–59% uptake reported in previous population-based studies.^6^ Low participation, exemplified by the 42% uptake, may mute the population-level benefits of CRC screening, leading some to perceive it as disappointing. Nevertheless, it is crucial to emphasize that the individual-level benefit for participants, which is closer to the NordICC trial’s adjusted per-protocol results, is more reassuring and reaffirms the effectiveness of the test. It is important to highlight that individuals who choose not to participate in screening do not receive any screening benefits, underlining the value of screening. Moreover, individuals should be aware that more favorable outcomes may be expected in the long term, especially beyond 15 years of follow-up, and that larger benefits could be achieved with repeated 10-yearly colonoscopy screening, as recommended in the United States. In conclusion, our findings show that NordICC trial results are consistent with anticipated mortality reductions from screening colonoscopy, and that with further follow-up higher benefits may be realized, especially in the NordICC’s per-protocol analyses.

## Supplementary Material

1

## Figures and Tables

**Figure 1.**
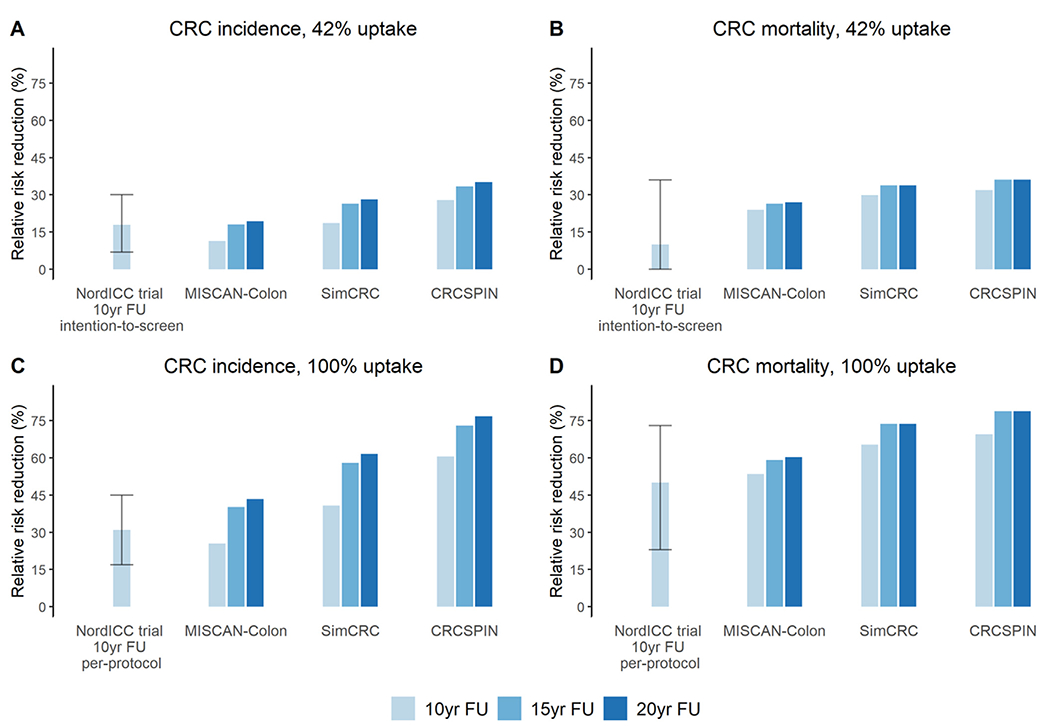
Relative risk reductions in CRC incidence (*A*, *C*) and CRC mortality (*B*, *D*) compared to no screening for 2 different uptake scenarios (42% and 100% uptake) and 3 different follow-up durations (10, 15, and 20 years). CRCSPIN, Colorectal Cancer Simulated Population Model for Incidence and Natural History; FU, follow-up; MISCAN-Colon, Microsimulation Screening Analysis Colorectal Cancer; SimCRC, Simulation model of Colorectal Cancer.

## Data Availability

Model results and R code for figures are available upon request.

## Abbreviations used in this paper:

CRC: colorectal cancer
NordICC: Northern European Initiative on Colorectal Cancer
