# Supplementary material for: NordICC Trial Results in Line With Expected Colorectal Cancer Mortality Reduction After Colonoscopy: A Modeling Study
Source: Gastroenterology. Author manuscript; Available in PMC 2024 Mar 25. (PMC10962655; doi:10.1053/j.gastro.2023.06.035)
Supplement: 1 [file NIHMS1973091-supplement-1.pdf]

Supplementary Materials

Model Descriptions

This study used 3 independently developed microsimulation models: Microsimulation Screening Analysis for Colorectal Cancer (MISCAN-Colon), Simulation Model of Colorectal Cancer (SimCRC), and Colorectal Cancer Simulated Population Model for Incidence and Natural History (CRCSPIN). Each model has a natural history and a screening component, summarized below.

Natural History Component

All models describe the natural history of CRC in an average-risk unscreened population. We assumed that all CRC develops through the adenoma-carcinoma pathway and that simulated persons are free of diagnosed CRC until screening in 2012. Each simulated individual can develop 1 or more colorectal lesions. Lesions may proceed through 3 phases: a noninvasive adenoma phase, a preclinical cancer phase, and a clinical cancer phase. Persons may die of other causes at any time.

Each model's natural history component was initially calibrated to Surveillance, Epidemiology, and End Results (SEER) data for the period 1975–1979. To adjust the models for CRC risk differences between SEER 1975–1979 and Norway and Poland, we compared CRC incidences in the countries. The magnitude of the difference was estimated by the ratio of CRC incidence in Norway and Poland between 2009 and 2010 relative to SEER data from 1975–1979 (Supplementary Table 1). We assumed that the decreased risk arises from changes in adenoma onset, not from slower progression of adenomas to CRC.

Screening Component

Screening will alter some of the simulated life histories: some cancers will be prevented by the detection and removal of adenomas; other cancers will be detected in an earlier stage with a more favorable survival. The ability of a test to detect lesions depends on its sensitivity. These sensitivities are lesion based. We assumed a colonoscopy sensitivity of 0.75, 0.85, 0.95, and 0.95 for adenomas of 1 to $<$6 mm, adenomas of 6 to $<$10 mm, adenomas of $\geq$10 mm, and CRC, respectively. We assumed the same sensitivities for surveillance colonoscopy as for screening colonoscopy.

Moreover, we assumed that individuals with an adenoma detected undergo colonoscopy surveillance according to the Multi-Society Task Force guidelines.[5] We assumed persons with adenoma findings are perfectly adherent with the surveillance colonoscopy schedules. Additionally, we assumed that persons in whom adenoma(s) have been detected remain on surveillance until age 85 years, provided that no adenomas are detected at the last surveillance colonoscopy. If adenomas are detected, then surveillance continues according to the clinical findings at the last colonoscopy until the person has a colonoscopy with no adenomas detected.

Outcomes

Outcomes were simulated for 10 different birth cohorts (birth years ranging from 1948 to 1957). For each cohort, we simulated 2 strategies: (1) no screening and (2) once-only colonoscopy in 2012 with 100% adherence and surveillance for people with adenomas detected. The results of the different strategies were consolidated afterward in the postprocessing based on the screening participation of the trial participants.[3] All outcomes were tallied by year from 2012 onward. The primary outcomes included the number of CRC cases and CRC deaths.

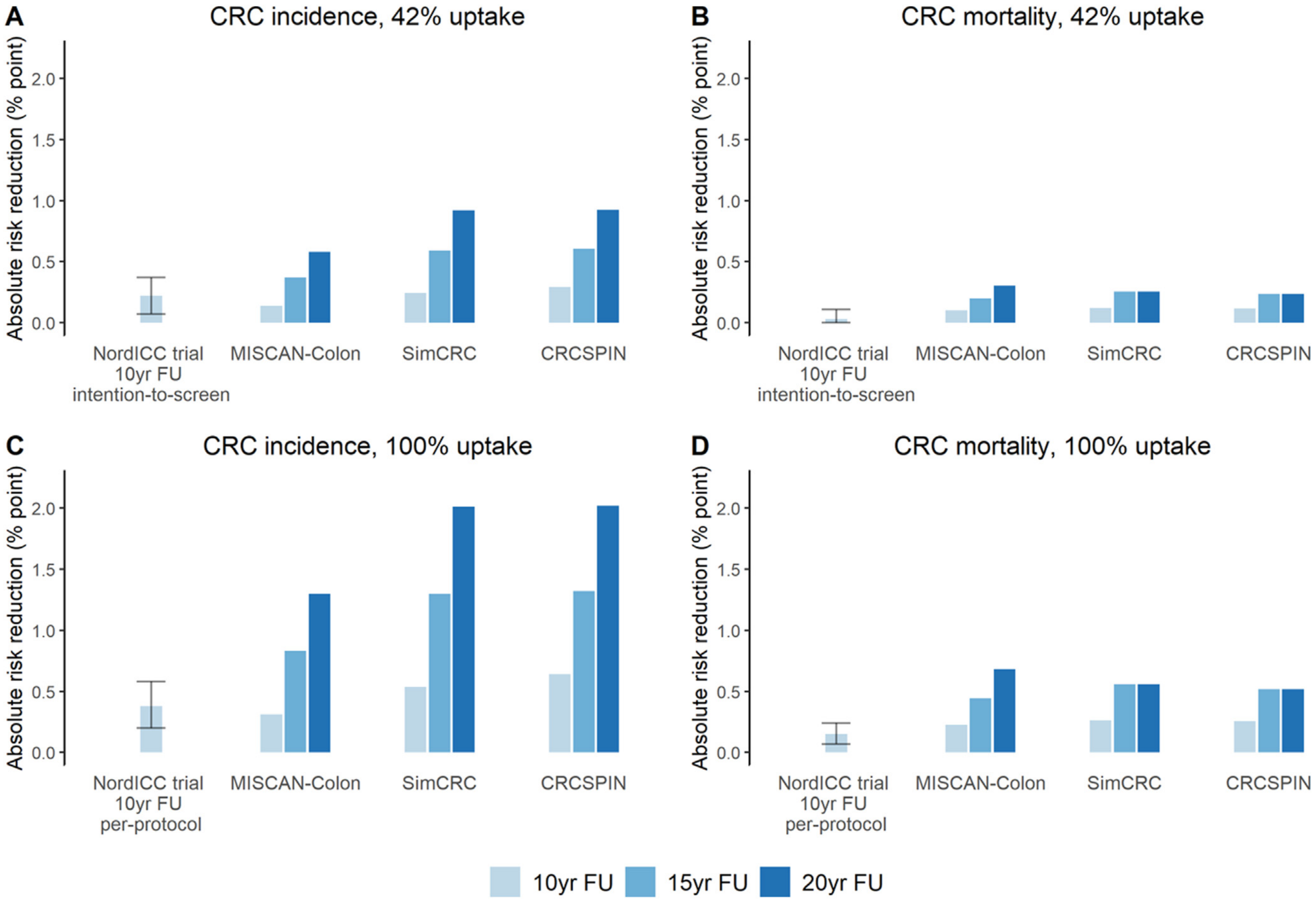

Supplementary Figure 1. Absolute risk reductions in CRC incidence (*A*, *C*) and CRC mortality (*B*, *D*) compared to no screening for 2 different uptake scenarios (42% and 100% uptake) and 3 different follow-up durations (10, 15, and 20 years). CRCSPIN, Colorectal Cancer Simulated Population Model for Incidence and Natural History; FU, follow-up, MISCAN-Colon, Microsimulation Screening Analysis Colorectal Cancer; SimCRC, Simulation Model of Colorectal Cancer.

Supplementary Table 1. CRC Age-Adjusted (World Population) Incidence Rates for SEER 1975–1979, Norway 2009–2010, and Poland 2009–2010 and CRC Incidence Rate Ratios Among Norway and Poland 2009–2010 vs SEER 1975–1979

Period	CRC cases per 100,000	CRC rate ratio
SEER, 1975–1979	39.3	1
Norway, 2009–2010	39.8	1.0
Poland, 2009–2010	25.3	0.6

NOTE. Source for Poland and Norway data: European Cancer Information System (https://ecis.jrc.ec.europa.eu).
